# Effects of plant growth regulators on the contents of rutin, hyperoside and quercetin in *Hypericum attenuatum* Choisy

**DOI:** 10.1371/journal.pone.0285134

**Published:** 2023-05-03

**Authors:** Rui Song, Yunrui Xia, Zhe Zhao, Xing Yang, Nanyi Zhang

**Affiliations:** Jilin Provincial Key Laboratory of Tree and Grass Genetics and Breeding, College of Forestry and Grassland Science, Jilin Agricultural University, Changchun, Jilin Province, China; Bangabandhu Sheikh Mujibur Rahman Agricultural University, BANGLADESH

## Abstract

To explore the accumulation of rutin, hyperoside and quercetin in *Hypericum attenuatum* Choisy under treatment with different plant growth regulators, 100 mg/L, 200 mg/L and 300 mg/L cycocel, 100 mg/L, 200 mg/L and 300 mg/L mepiquat chloride and 1 mg/L, 2 mg/L and 3 mg/L naphthalene acetic acid were foliage sprayed on *Hypericum attenuatum* Choisy plants at the early growth stage. We sampled and determined the important flavonoid contents at the flowering stage. The results showed that the three plant growth regulators had different effects on the accumulation of rutin, hyperoside and quercetin in the leaves, stems and flowers of *Hypericum attenuatum* Choisy at the flowering stage. After spraying 1 mg/L naphthalene acetic acid at the early growth stage, the rutin contents in the leaves, stems and flowers increased by approximately 60.33%, 223.85% and 192.02%, respectively (*P* < 0.05). Spraying 100 mg/L mepiquat chloride increased the hyperoside contents in the leaves and flowers by approximately 7.77% and 12.87%, respectively (*P* < 0.05). Spraying 2 mg/L naphthalene acetic acid significantly increased the quercetin contents in the flowers and leaves by approximately 95.62% and 47.85%, respectively (*P* < 0.05). Therefore, at the early growth stage, spraying 1 mg/L naphthalene acetic acid significantly increased rutin content, spraying 100 mg/L mepiquat chloride significantly increased hyperoside content, and spraying 2 mg/L naphthalene acetic acid significantly increased quercetin content in *Hypericum attenuatum* Choisy. In conclusion, the accumulation of flavonoids in *Hypericum attenuatum* Choisy was regulated by plant growth regulators.

## Introduction

*Hypericum attenuatum* Choisy, also known as Gangshan whip and small tea leaves, is a perennial herb of the genus Hypericum in the Garcinia family [1]. It is highly adaptable, drought-tolerant, cold-tolerant, light-loving, has no strict soil fertility requirements and is widely distributed in eastern Siberia and the Far East countries of China, Korea, Mongolia and Japan. Hypericum has a variety of pharmacological activities and has been widely used in folk medicine [2]. Chinese folk medicine often uses autumn harvest drying for hemostasis, analgesia, treatment of injuries and so on. Modern pharmacological studies have also found that it has promising applications in the treatment of tumors, depression, arrhythmogenic heart disease and diabetes [3–6].

Some scholars have extracted, isolated and identified flavonoids, such as hyperoside, quercetin, isoquercetin, queritroside, and rutin, from *Hypericum attenuatum* Choisy [7–9]. Pharmacological and clinical application studies have also confirmed that these compounds have good effects on the treatment of various diseases [10]. Studies found that the combination of hyperoside and quercetin effectively inhibited cancer cell growth and proliferation [11]. Hyperoside significantly inhibits three kinds of human-derived cancer cells, activates the dopamine receptor-mediated central nervous system and effectively reduces the immobility time of mice during forced swimming [12, 13]. Rutin significantly inhibits the activity of lung and colon cancer cells, reduced the production of superoxide in colon cancer cells, and affects the adhesion and migration of lung and colon cancer cells [14]. In addition, some scholars have found that rutin reduced fasting and non-fasting blood glucose levels; at the same time, reduced the oxidative stress of kidney tissue, prevented the formation of glycosylated products and alleviated diabetic kidney injury [15, 16]. Studies of quercetin have shown that quercetin can significantly reduce the number of premature ventricular contractions, reduce the replication rate of HCV and block virus fusion [17–19].

Flavonoids are the active ingredients in most Chinese medicinal materials, and one of the main focuses of pharmaceutical research [20]. Flavonoids are one of the main active components in *Hypericum attenuatum* Choisy. The synthesis and accumulation of flavonoids in plants is influenced by a number of factors [21, 22], including plant hormones. Plant growth regulators can be used to regulate plant hormone levels and thereby affecting the flavonoid content in *Hypericum attenuatum* Choisy.

Cycocel (CCC) as an antagonist of gibberellin, which prevents plants from producing flavonoids [23]. Previous studies have found gibberellin could participate in phenylpropane metabolism and regulate the accumulation of flavonoids such as anthocyanins [24–27]. In addition, cycocel could regulate source-sink balance and carbohydrate metabolism balance in plants [28]. Mepiquat chloride (MCD) is a synthetic antigibberellin analogue, which has effects on the flavonoid content of plants being similar to those of cycocel. Studies have found that gibberellin could delay the accumulation of flavonoids in rice roots, and was negatively correlated with flavonoid content [29, 30]. As an antagonist of gibberellin, MCD may inhibit the negative effect of gibberellin on the accumulation of flavonoids in plants. However, the effect of mepiquat chloride treatment on the flavonoid content of the underground parts of plants was not consistent with that of cycocel. Naphthalene acetic acid (NAA) is a synthetic auxin analogue, that promotes plant growth and rooting cuttings, induces flowering and inhibits fruit drop in agricultural production [31]. Previous studies have found that auxin is involved in the transport of flavonoids in plants and has a certain relationship with flavonoid synthesis [32–34].

At present, studies on the regulation of the content of the active ingredients of *Hypericum attenuatum* Choisy are relatively rare. To date, no studies on the effects of plant growth regulators on the flavonoid contents of *Hypericum attenuatum* Choisy have been reported. Plant growth regulators have been proven by many studies to have a beneficial effect on increasing the flavonoid content of plants. The content of plant endogenous hormones correlates with the content of flavonoids in plants [21, 35, 36], and growth regulators affect the changes in endogenous hormone content in plants to a certain extent [37–39]. At the same time, plant growth regulators also affect the expression of key enzymes in the synthesis of flavonoids and control the synthesis of flavonoids [40, 41]. Therefore, this experiment was conducted to investigate the effects of different concentrations of cycocel, mepiquat chloride and naphthalene acetic acid on the contents of hyperoside, rutin, and quercetin in *Hypericum attenuatum* Choisy to provide a theoretical basis for the rational utilization and conservation of *Hypericum attenuatum* Choisy.

## Materials and methods

### Plant materials and test site overview

Triennial *Hypericum attenuatum* Choisy were selected as plant material for the experiment; the seeds were collected from the Zuojia Nature Reserve in Jilin Province (N 44° 00 ’-44° 07’, E 126° 01 ’-126° 08’) in September 2017, and reviewed and identified by Professor Zhang, K. Q. of Jilin Agricultural Science and Technology University. The seeds were sown in March 2019 and planted in a pratacultural science experimental plot (N 43° 810433’, E 125° 410385’) of Jilin Agricultural University in Changchun, Jilin Province in April 2019.

Considering the spatial heterogeneity of the soil, a field with basically the same soil structure and fertility was chosen as the sample plot. The sample plot is located in the transition zone between the humid mountain in the east and the semi-arid plain in the west. It has a temperate continental semi-humid monsoon climate with hot, humid and rainy summer and long cold winter. Where the average annual temperature is 4.6°C, the average annual air humidity is 60%, the average annual precipitation is 600–700 mm, and the average summer temperature is 14.0°C-25.0°C. The sample plot is black soil with 15% soil water content, 7.25 mg/kg available phosphorus content, 24.7 mg/kg available nitrogen content, 0.4 ± 0.07 g/kg total phosphorus content, 2.14 ± 0.87 g/kg total nitrogen content and organic matter content 23.99 ± 10.91 g/kg. The soil carbon to phosphorus ratio, carbon to nitrogen ratio and Nitrogen to phosphorus ratio was 59.98, 11.21, and 5.35, respectively. The sample plot was divided into 10 test plots of 17 m × 5 m (length × width), each plot with three replicates, and 80 *Hypericum attenuatum* Choisy per replicate. In addition, there is a 1 m wide aisle between each replicate.

### Equipment and chemicals

An ultrasonic double-frequency cleaner (SB25-12, Ningbo Xinzhi Biotechnology Co., Ltd.), rotary evaporator (RE-52AA, Shanghai Yarong Biochemical Instrument Factory), and high-performance liquid chromatograph (AT-20, Shimadzu, Japan) were used for the experiments. Cycocel, α-naphthalene acetic acid, rutin standard, and quercetin standard were purchased from Beijing Solaibao Technology Co., while mepiquat chloride and hyperoside standards were purchased from Shanghai Yuanye Biotechnology Co.

### Experimental design

Ten treatments were designed in this experiment: 100 mg/L, 200 mg/L and 300 mg/L of cycocel, labeled 100 C, 200 C and 300 C, respectively; 100 mg/L, 200 mg/L and 00 mg/L of mepiquat chloride, labeled 100 S, 200 S and 300 S, respectively; and 1 mg/L, 2 mg/L and 3 mg/L of naphthalene acetic acid, labeled 1 NAA, 2 NAA and 3 NAA, respectively. The blank control group was sprayed with equal amounts of water, labeled CK.

When the most of the resume growth seedlings were 10 or more pairs of leaves (the early growth stage of *Hypericum attenuatum* Choisy), the 10 test plots were sprayed simultaneously. The solution (2 mL) was sprayed per plant, covering the foliage evenly, and it took 3 hours to spray all test plots. Twice in total, and an interval of 8–10 days. Importantly, to ensure that the day, when drug spraying, was no wind and no rainfall for 24 h. The first spray was applied on 11 May and the second on 19 May.

Considering the flavonoid content of *Hypericum attenuatum* in the flowering stage was highest [42], the samples were collected from each replicate group using the five-point sampling method in the flowering stage (July 1), for a total of 75 plants per treatment. Then the stems, leaves and flowers were separated from the collected samples and labeled separately. Placed samples in a drying oven for drying at 50°C to a constant weight [43, 44], then fully ground into powder and screened (40 mesh) to make test sample powder. The powdered samples were placed in self-sealing bags and stored in a refrigerator at -20°C.

### Flavonoid content measurement

#### Preparation of test materials

A total of 1 g powder sample was precisely weighed and combined with 40 mL acetone (liquid‒solid ratio: 1:40 g/mL) in a 50 mL centrifuge tube and shaken well. Then the solution was ultrasonic extracted three times at 50°C for 40 min (ultrasonic cleaner power 200 W, vibration frequency 40 kHz). After the extracting solution cooling, the extracting solution was centrifuged for 10 min (4000 rpm). The supernatant was collected and rotary evaporated to dryness at 50°C in a water bath. Then using an appropriate amount of chromatographic methanol to dissolve the evaporated, and fixed into a 5 mL brown volumetric flask. The extracting samples were filtered with a 0.22 μm microporous membrane. The filtrate was packed into a brown penicillin bottle, sealed and stored in a refrigerator at -20°C.

#### Preparation of reference substance

Masses of 6.0 mg standard rutin, 5.8 mg standard hyperoside and 10.4 mg standard quercetin were weighed precisely in a beaker. A proper amount of methanol was added to dissolve the flavonoid standards, and the volume was kept at 25 mL. The filtrate was filtered through a 0.22 μm microporous membrane and placed into a brown penicillin bottle.

#### The chromatographic conditions

The flavonoid content of *Hypericum attenuatum* Choisy were determined using the method of Li et al [45]. with improvements. A high-performance liquid chromatograph/HPLC (1525 detector pump, 2487 dual-wavelength UV detector, Empower chromatographic workstation) with a chromatographic column (4.6 mm×250 mm, 2.5 μm) was used to assay analysis. Mobile phase A was acetonitrile, and mobile phase B was 0.2% phosphoric acid solution. The gradient was as follows: 0–10 min 15% A, 85% B; 10–30 min 20% A, 80% B; 30–45 min 25% A, 75% B; 45–60 min 27% A, 73% B; and 60–80 min 15% A, 85% B. The column temperature was 35°C, the detection wavelength was 270 nm, the flow rate was 0.8 mL/min, and the injection volume was 20 μL.

#### Preparation of standard curves

2 μL, 4 μL, 6 μL, 10 μL and 20 μL of the standard solution were precisely absorbed and examined by HPLC. The standard curves were made by taking the injection quantity (μL) as abscissa X and the peak area value (A) as ordinate Y. For rutin, *$Y_{1}=\left(5.99421e-0.07\right)\;x_{1\;}+\;\left(-3.132329e-0.02\right),\;R_{1}^{2}=0.99941$*; For hyperoside,$\;Y_{2}=\left(5.678987e-0.07\right){\;x}_{2}\;+\;\left(-9.233763e-0.02\right),\;R_{2}^{2}=0.9994344$; for quercetin,$\;Y_{3}=\left(3.332914e-0.07\right){\;x}_{3}\;+\;\left(-3.978964e-0.02\right),\;R_{3}^{2}=0.9987463$. R_1_^2^ = 0.99941, R_2_^2^ = 0.9994344, and R_3_^2^ = 0.9987463 showed a good linear relationship between peak area and content.

#### Test sample determination

Under these conditions, HPLC was used to determine the rutin, hyperoside and quercetin content of the *Hypericum attenuatum* Choisy test solution.

### Data processing

The data were analyzed using SPSS 22.0 (Statistical Product and Service Solutions 22.0 for Windows, USA). Analysis of variance (ANOVA) was determined using LSD, and significance was accepted at *P* < 0.05. All analyses and determinations were conducted in triplicate, and the results are expressed as the means of triplicate measurements ± standard deviation. Excel software was used for data collation, and Sigmaplot 14.0 was used to plotting.

## Results

### Variation in rutin content in *Hypericum attenuatum* Choisy

**Fig 1:**
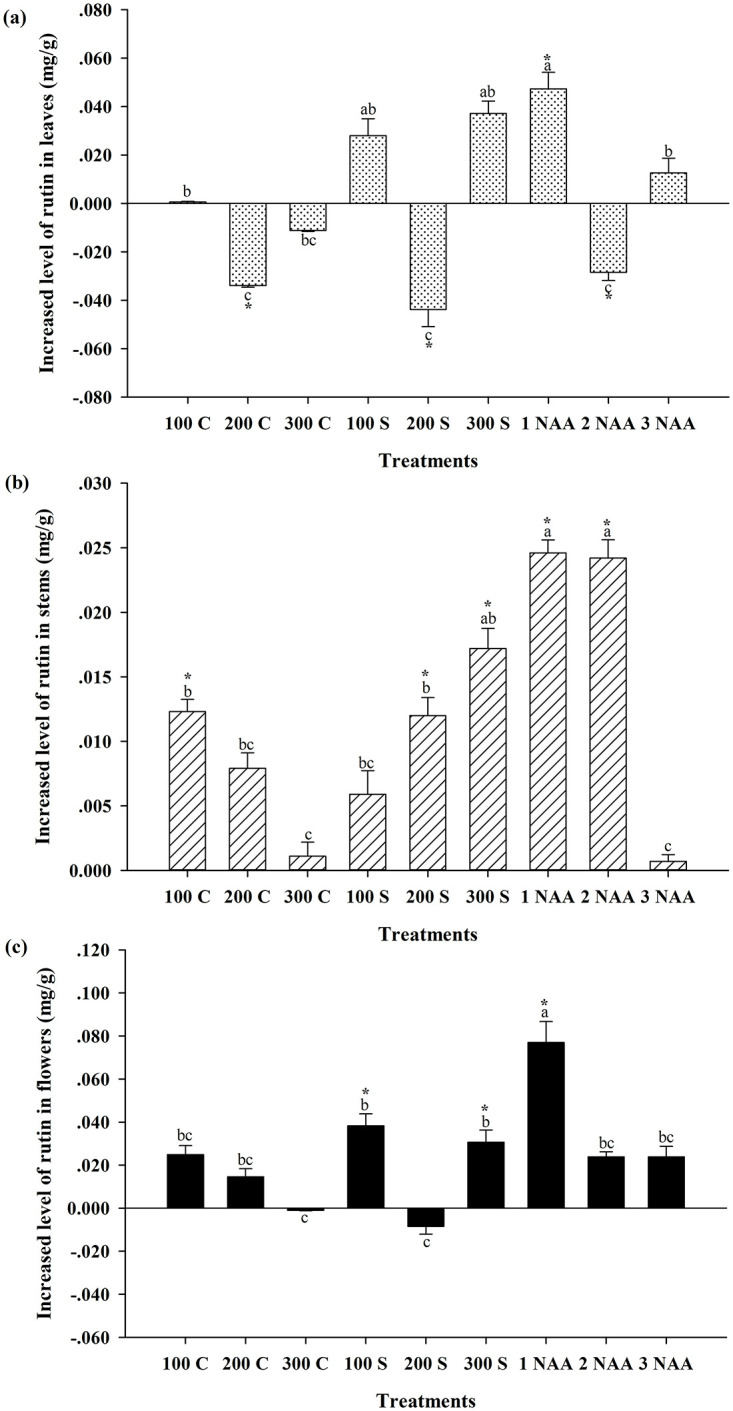
Effects of plant growth regulators on the content of rutin of *Hypericum attenuatum* Choisy. 100 C = 100 mg/L cycocel, 200 C = 200 mg/L cycocel, 300 C = 300 mg/L cycocel, 100 S = 100 mg/L mepiquat chloride, 200 S = 200 mg/L mepiquat chloride, 300 S = 300 mg/L mepiquat chloride, 1 NAA = 1 mg/L naphthalene acetic acid, 2 NAA = 2 mg/L naphthalene acetic acid, 3 NAA = 3 mg/L naphthalene acetic acid treatment, CK = Blank control group. a,b,c Means treatment groups without a common superscript are significantly different (*P* < 0.05). * Means the treatment group and the control group was significantly different (*P* < 0.05).

Analysis of variance (ANOVA) results for rutin content in *Hypericum attenuatum* Choisy showed the highest rutin content in the leaves and the lowest rutin content in the stems at the flowering stage (Fig 1). Compared with the blank control, spraying 200 mg/L CCC, 200 mg/L MCD, and 2 mg/L NAA at the early growth stage significantly reduced the content of rutin in leaves at the flowering stage (*P* < 0.05), resulting in a decrease of approximately 43.24%, 55.87%, and 36.22% of rutin in leaves, respectively. However, 1 mg/L NAA sprayed at the early growth stage significantly increased rutin content in leaves by approximately 60.33% (*P* < 0.05) to 0.126 ± 0.014 mg/g (Table 1). Although 100 mg/L and 300 mg/L MCD sprayed at the early stage of growth also increased rutin content in leaves, the difference from the control group was not significant (*P* > 0.05).

**Table 1:** Content of rutin in various parts of Hypericum attenuatum Choisy treated with plant growth regulator.

Treatments	Rutin content in leaves (mg/g)	Rutin content in stems (mg/g)	Rutin content in flowers (mg/g)
CK	0.078±0.007^b^	0.011±0.001^c^	0.040±0.003^c^
100C	0.079±0.002^b^	0.023±0.004^b^	0.065±0.008^b^^c^
200C	0.045±0.001^c^	0.019±0.005^b^^c^	0.055±0.007^b^^c^
300C	0.067±0.000^b^^c^	0.012±0.001^c^	0.039±0.000^c^
100S	0.106±0.007^a^^b^	0.017±0.002^b^^c^	0.078±0.011^b^
200S	0.035±0.007^c^	0.023±0.006^b^	0.032±0.007^c^
300S	0.116±0.020^a^^b^	0.028±0.002^a^^b^	0.071±0.011^b^
1NAA	0.126±0.014^a^	0.036±0.001^a^	0.117±0.020^a^
2NAA	0.050±0.003^c^	0.035±0.006^a^	0.064±0.002^b^^c^
3NAA	0.091±0.122^b^	0.012±0.001^c^	0.064±0.005^b^^c^

^a,b,c^Means in a column without a common superscript are significantly different (*P* < 0.05)

For the rutin content in stems at the flowering stage, spraying 100 mg/L CCC, 200 mg/L MCD, 300 mg/L MCD, 1 mg/L NAA, and 2 mg/L NAA at the early growth stage significantly increased the rutin content in stems (*P* < 0.05). Among those treatments, the greatest increase in rutin content in stems was achieved by spraying 1 mg/L or 2 mg/L NAA, and there was no significant difference between the two treatments (*P* > 0.05) with an average increase in rutin content in stems of approximately 223.85% compared to control. Spraying 300 mg/L MCD was the next most effective treatment, increasing the content of rutin in stems by approximately 157.80% compared to the control to 0.028 ± 0.002 mg/g (Table 1). The increase in rutin content in stems with the 100 mg/L CCC and 200 mg/L MCD treatments was lower, and the difference between the two treatments was not significant (*P* > 0.05). Compared to the control, the rutin content in stems was increased by 111.47% on average.

The results of rutin content in flowers of *Hypericum attenuatum* Choisy showed that spraying 100 mg/L MCD, 300 mg/L MCD, and 1 mg/L NAA at the early growth stage significantly increased the rutin content in flowers compared to the control group. The most significant increase in rutin content in flowers was achieved with 1 mg/g NAA (*P* < 0.05), which increased it by approximately 192.02% to 0.117 ± 0.020 mg/g (Table 1). There was no significant difference in rutin content in flowers between the 100 mg/g and 300 mg/g MCD treatments (*P* > 0.05), and the rutin content in flowers was increased by approximately 86.03% on average.

### Variation in hyperoside content in *Hypericum attenuatum* Choisy

**Fig 2:**
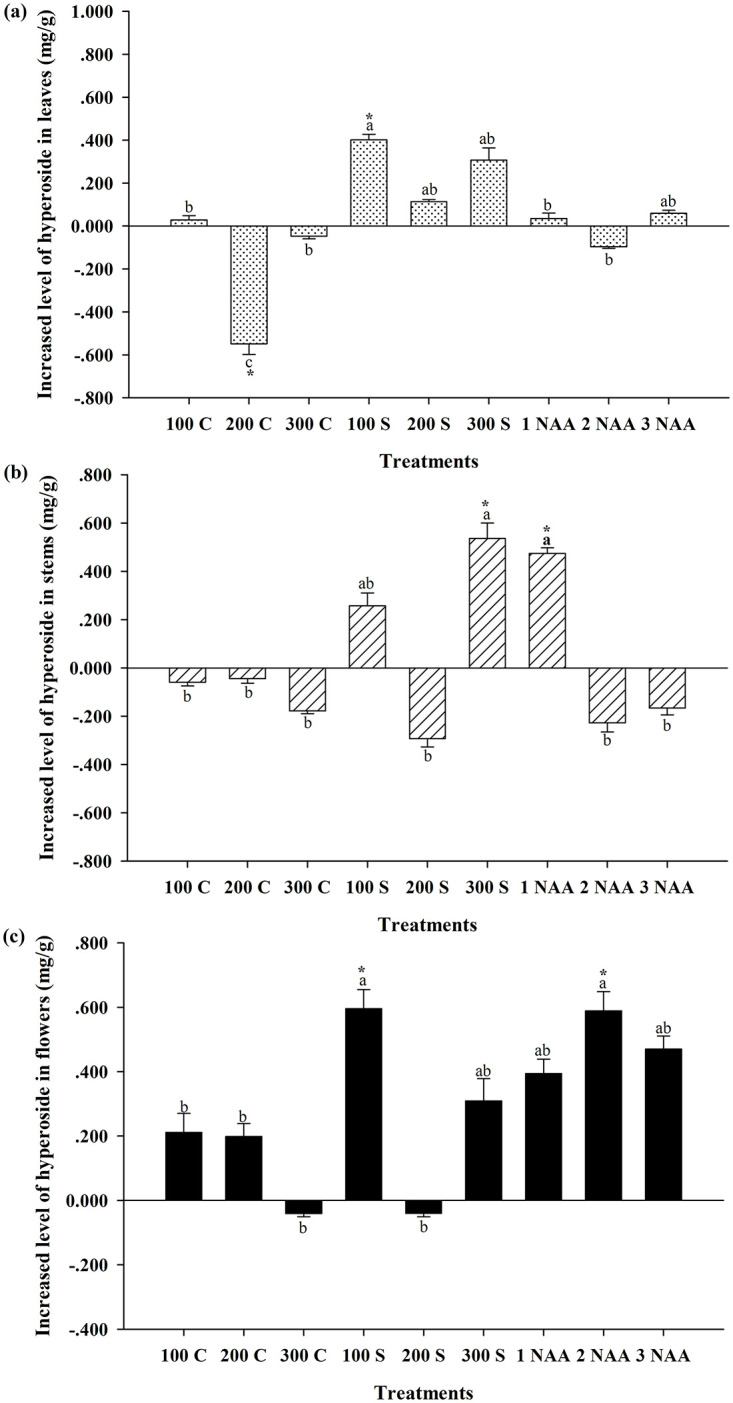
Effects of plant growth regulators on the content of hyperoside of *Hypericum attenuatum* Choisy. 100 C = 100 mg/L cycocel, 200 C = 200 mg/L cycocel, 300 C = 300 mg/L cycocel, 100 S = 100 mg/L mepiquat chloride, 200 S = 200 mg/L mepiquat chloride, 300 S = 300 mg/L mepiquat chloride, 1 NAA = 1 mg/L naphthalene acetic acid, 2 NAA = 2 mg/L naphthalene acetic acid, 3 NAA = 3 mg/L naphthalene acetic acid treatment, CK = Blank control group. a,b,c Means treatment groups without a common superscript are significantly different (*P* < 0.05). * Means the treatment group and the control group was significantly different (*P* < 0.05).

The study of hyperoside content in *Hypericum attenuatum* Choisy showed that the hyperoside content at the flowering stage was similar to that of rutin, with the highest hyperoside content in the leaves and the lowest in the stems (Fig 2). Compared to the control, spraying all plant growth regulators except for 200 mg/L CCC and 100 mg/L MCD at the early growth stage had no significant effect on the content of hyperoside in the leaves. The ANOVA results showed that spraying 200 mg/L CCC at the early growth stage significantly decreased the hyperoside content in the leaves (*P* < 0.05) by approximately 10.61% compared to the control. In contrast, spraying 100 mg/L MCD at the early growth stage significantly increased the hyperoside content in the leaves by approximately 7.77% (*P* < 0.05) to 5.569 ± 0.052 mg/g (Table 2).

**Table 2:** Content of hyperoside in various parts of *Hypericum attenuatum* Choisy treated with plant growth regulator.

Treatments	Hyperoside content in leaves (mg/g)	Hyperoside content in stems (mg/g)	Hyperoside content in flowers (mg/g)
CK	5.167±0.118^b^	2.054±0.019^b^	3.848±0.129^b^
100C	5.196±0.040^b^	1.994±0.015^b^	4.059±0.118^b^
200C	4.619±0.198^c^	2.010±0.193^b^	4.046±0.081^b^
300C	5.120±0.025^b^	1.875±0.011^b^	3.807±0.099^b^
100S	5.569±0.052^a^	2.311±0.267^a^^b^	4.443±0.177^a^
200S	5.281±0.019^a^^b^	1.761±0.175^b^	3.807±0.062^b^
300S	5.474±0.115^a^^b^	2.590±0.064^a^	4.157±0.137^a^^b^
1NAA	5.203±0.249^b^	2.528±0.023^a^	4.242±0.089^a^^b^
2NAA	5.071±0.014^b^	1.826±0.037^b^	4.437±0.179^a^
3NAA	5.227±0.056^a^^b^	1.887±0.028^b^	4.318±0.081^a^^b^

^a,b,c^Means in a column without a common superscript are significantly different (*P* < 0.05)

In contrast to the effect of plant growth regulators on the content of hyperoside in leaves at the flowering stage, only spraying 300 mg/L MCD and 1 mg/L NAA at the early growth stage significantly influenced the hyperoside content in stems (*P* < 0.05). Spraying either 300 mg/L MCD or 1 mg/L NAA at the early growth stage significantly increased the content of hyperoside in the stems (*P* < 0.05), and there was no significant difference between the two treatments (*P* > 0.05), with an increased hyperoside content in stems of 24.62% on average.

Compared to the control group, the levels of hyperoside in flowers treated with all growth regulators except for 100 mg/L MCD and 2 mg/L NAA were different but did not reach significant levels (*P* > 0.05). Spraying 100 mg/L MCD and 2 mg/L NAA significantly increased the hyperoside content in flowers (*P* < 0.05), and there was no significant difference between the two treatments (*P* > 0.05), with an increased hyperoside content in flowers of 12.87% on average.

### Variation in quercetin content in *Hypericum attenuatum* Choisy

**Fig 3:**
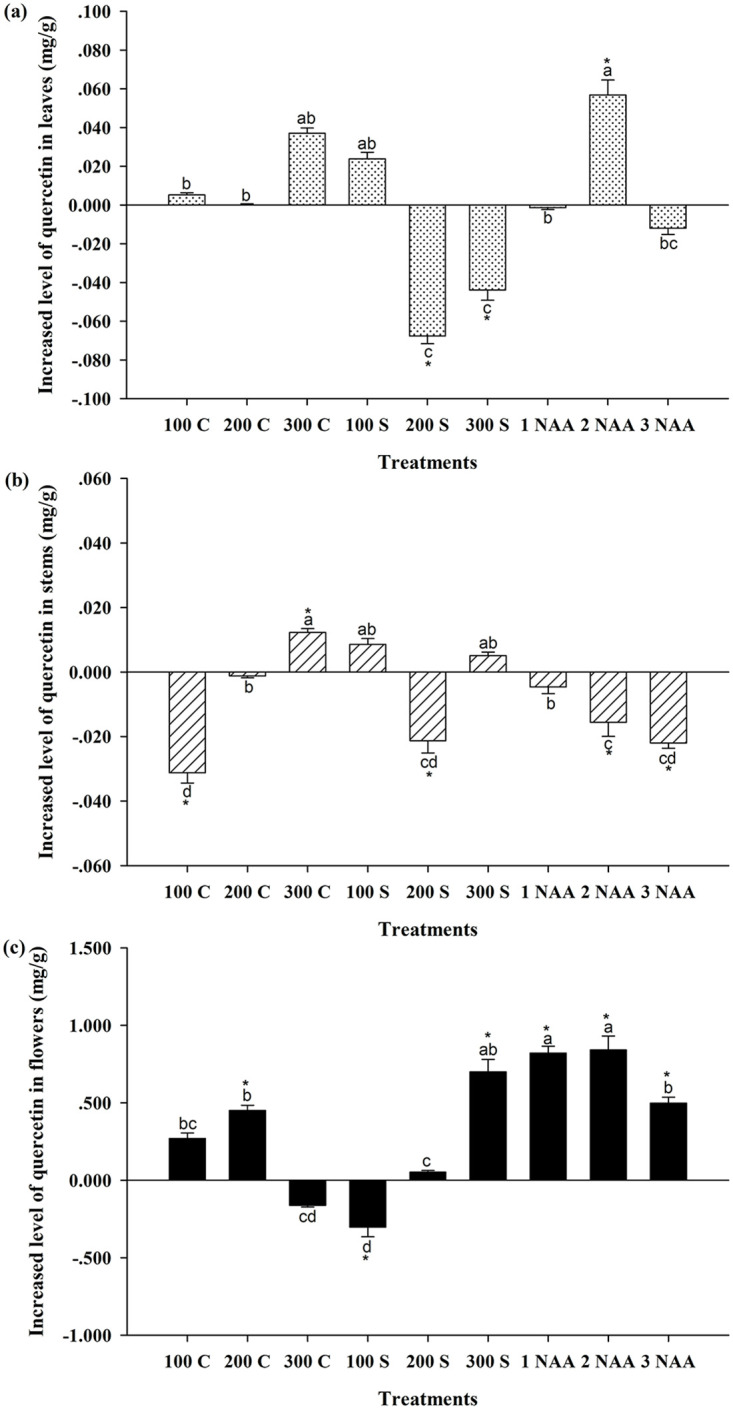
Effects of plant growth regulators on the content of quercetin of *Hypericum attenuatum* Choisy. 100 C = 100 mg/L cycocel, 200 C = 200 mg/L cycocel, 300 C = 300 mg/L cycocel, 100 S = 100 mg/L mepiquat chloride, 200 S = 200 mg/L mepiquat chloride, 300 S = 300 mg/L mepiquat chloride, 1 NAA = 1 mg/L naphthalene acetic acid, 2 NAA = 2 mg/L naphthalene acetic acid, 3 NAA = 3 mg/L naphthalene acetic acid treatment, CK = Blank control group. a,b,c Means treatment groups without a common superscript are significantly different (*P* < 0.05). * Means the treatment group and the control group was significantly different (*P* < 0.05).

The data analysis results of quercetin content of *Hypericum attenuatum* Choisy at the flowering stage showed that (Fig 3), compared to the control, the quercetin content in leaves sprayed with 200 mg/L MCD, 300 mg/L MCD, and 2 mg/L NAA was significantly different, while there were no significant differences in quercetin content in leaves after the other treatments (*P* > 0.05). Spraying 2 mg/L NAA significantly increased the quercetin content in leaves by approximately 47.85% to 0.176 ± 0.016 mg/g (Table 3). However, spraying 200 mg/L or 300 mg/L MCD significantly reduced the quercetin content in leaves, and there was no significant difference between the two treatments (*P* > 0.05). Compared to the control, the average reduction in quercetin content was approximately 46.97%.

**Table 3:** Content of quercetin in various parts of *Hypericum attenuatum* Choisy treated with plant growth regulator.

Treatments	Quercetin content in leaves (mg/g)	Quercetin content in stems (mg/g)	Quercetin content in flowers (mg/g)
CK	0.119±0.016^b^	0.056±0.006^b^	0.870±0.100^c^
100C	0.124±0.023^b^	0.025±0.003^d^	1.139±0.143^b^^c^
200C	0.119±0.013^b^	0.055±0.006^b^	1.321±0.033^b^
300C	0.156±0.003^a^^b^	0.068±0.001^a^	0.707±0.011^c^^d^
100S	0.143±0.007^a^^b^	0.065±0.002^a^^b^	0.567±0.063^d^
200S	0.051±0.004^c^	0.035±0.004^b^^d^	0.922±0.103^c^
300S	0.075±0.005^c^	0.061±0.001^a^^b^	1.570±0.158^a^^b^
1NAA	0.117±0.018^b^	0.052±0.002^b^	1.691±0.044^a^
2NAA	0.176±0.016^a^	0.041±0.004^c^	1.711±0.090^a^
3NAA	0.107±0.006^b^^c^	0.034±0.002^c^^d^	1.368±0.037^b^

^a,b,c^Means in a column without a common superscript are significantly different (*P* < 0.05)

Compared with the control group, spraying 300 mg/L CCC at the early growth stage significantly increased the quercetin content in stems by approximately 21.93% to 0.068 ± 0.001 mg/g (Table 3). In contrast, spraying 100 mg/L CCC, 200 mg/L MCD, 2 mg/L NAA, and 3 mg/L NAA negatively affected quercetin content in stems at the flowering stage and significantly reduced quercetin content in stems by approximately 55.61%, 37.97%, 27.81%, and 39.22%, respectively. Among those treatments, the most significant inhibitory effect on quercetin content in stems was achieved by spraying with 100 mg/L CCC.

Quercetin content in flowers was more sensitive to the effects of growth regulators than rutin or hyperoside in *Hypericum attenuatum* Choisy. In contrast to the effects of the 100 mg/L MCD treatment on rutin and hyperoside, it had a negative effect on the quercetin content in flowers compared to the control, which resulted in a significant reduction of approximately 34.81% in the quercetin content in flowers (*P* < 0.05). However, spraying 200 mg/L CCC, 300 mg/L MCD, 1 mg/L NAA, 2 mg/L NAA, and 3 mg/L NAA significantly increased the quercetin content in flowers (*P* < 0.05). Compared to the control, spraying 1 mg/L or 2 mg/L NAA increased the quercetin content in flowers most significantly, and there was no significant difference between the two treatments (*P* > 0.05), with an average increase of approximately 95.62%. Spraying 300 mg/L MCD increased the quercetin content in flowers to the second highest level, with an increase of approximately 80.55%. Spraying 3 mg/L NAA and 200 mg/L CCC showed the lowest level of increase in quercetin content in flowers, with an average increase of approximately 54.58%.

## Discussion

In terms of the effect of the plant growth regulator treatments on the rutin content of *Hypericum attenuatum* Choisy, the distribution of rutin content was different at the flowering stage; the rutin content was the highest in leaves, the second highest in flowers, and the lowest in stems. This is consistent with the results of Liu et al [46]. Spraying 1 mg/L NAA at the early growth stage increased the rutin content in leaves, stems and flowers at the flowering stage by 60.33%, 223.85%, and 192.02%, respectively. However, although 300 mg/L MCD sprayed at the early growth stage increased rutin content in stems and flowers, there was no significant gain in rutin content in leaves at the flowering stage, so the level of increase was lower than that achieved with the 1 mg/L NAA treatment. Spraying 100 mg/L MCD at the early growth stage increased rutin content in flowers but had no effect on rutin content in leaves or stems. In addition, spraying 2 mg/L NAA or 200 mg/L MCD at the early growth stage increased rutin content in stems, did not affect rutin content in flowers, and significantly reduced rutin content in leaves. The order of rutin content in different parts of *Hypericum attenuatum* Choisy was leaves > flowers > stems. Therefore, spraying 1 mg/L NAA at the early growth stage resulted in the most significant increase in rutin content at the flowering stage. In addition, the plant growth regulators did not have any positive effect on the rutin content of *Hypericum attenuatum* Choisy at leaf expansion. Over time, the increase in rutin content in *Hypericum attenuatum* Choisy at the vigorous growth and flowering stages induced by the 1 mg/L NAA treatment became apparent, and the effect was remarkable.

In this study have found significant variability in hyperoside content in different parts of *Hypericum attenuatum* Choisy, with the highest hyperoside content in leaves and the lowest in stems. In terms of hyperoside content in *Hypericum attenuatum* Choisy, spraying 2 mg/L NAA at the early growth stages increased the hyperoside content in flowers; however, there was no significant improvement in the hyperoside content in the leaves at the flowering stage. Although spraying 300 mg/L MCD and 1 mg/L NAA increased the hyperoside content in the stems of *Hypericum attenuatum* Choisy at the flowering stage, by comparing the absolute values of hyperoside in different parts of *Hypericum attenuatum* Choisy, we found that the hyperoside content in the stems was much lower than that in the leaves and flowers, at only half their content. Thus, the increases in hyperoside content with the 300 mg/L MCD and 1 mg/L NAA treatments were significantly lower than that of the 2 mg/L NAA treatment. However, spraying 100 mg/L MCD at the early growth stage significantly increased the hyperoside content in both the leaves and flowers at the flowering stage by 7.77% and 12.87%, respectively, and had no inhibitory effect on the levels of hyperoside in the stems at the flowering stage. This treatment showed the greatest increase in the hyperoside content of *Hypericum attenuatum* Choisy at the flowering stage. In contrast, spraying 200 mg/L CCC at the early growth stage significantly reduced the hyperoside content in the leaves at the flowering stage and had no positive effect on the content of hyperoside in the flowers or stems; thus, that treatment could effectively reduce the hyperoside content of *Hypericum attenuatum* Choisy at the flowering stage. In conclusion, spraying 100 mg/L MCD at the early growth stage was the most effective treatment for increasing the hyperoside content of *Hypericum attenuatum* Choisy at the flowering stage; spraying 200 mg/L CCC reduced the hyperoside content of *Hypericum attenuatum* Choisy at the flowering stage.

In contrast to the rutin and hyperoside contents, the quercetin content in *Hypericum attenuatum* Choisy was highest in flowers, second highest in leaves and lowest in stems at the flowering stage. Spraying 2 mg/L NAA at the early growth stages produced the highest level of gain in quercetin content in flowers and leaves, with 95.62% and 47.85% increases, respectively, but decreased quercetin content in stems. By comparison, it was found that the quercetin content in flowers and leaves was much higher than that in stems. The lowest level of quercetin inhibition was 27.81% in stems at the flowering stage with 2 mg/L NAA. Therefore, spraying 2 mg/L NAA at the early growth stages could improve the quercetin content in *Hypericum attenuatum* Choisy at the flowering stage. Spraying 1 mg/L NAA and 200 mg/L CCC only increased the quercetin content in flowers but had no significant effect on that in leaves or stems at the flowering stage. Although spraying 300 mg/L MCD increased quercetin content in flowers, it decreased quercetin content in leaves at the flowering stage. Spraying 3 mg/L NAA increased quercetin content in flowers, but had no significant effect on quercetin content in leaves and reduced quercetin content in stems at the flowering stage. Spraying 300 mg/L CCC increased the quercetin content in stems, but had no effect on the quercetin content in flowers or leaves at the flowering stage. The quercetin content in the flowers and leaves was much higher than that in stems. Thus spraying 300 mg/L CCC did not significantly increase the quercetin content in the plants. In conclusion, spraying 2 mg/L NAA at the early growth stages could result in the highest level of quercetin content gain in *Hypericum attenuatum* Choisy at the flowering stage.

Naphthalene acetic acid is a plant growth promoter that has the ability to promote plant growth and induce plant flowering, among other effects [31]. In addition, naphthalene acetic acid also involved in the transport of flavonoids [32]. In this study found that spraying medium concentrations of naphthalene acetic acid was the most effective treatment for increasing the quercetin content in *Hypericum attenuatum* Choisy, with higher concentrations tending to reduce quercetin content. In addition, during the experimental study, 2 mg/L naphthalene acetic acid treatment had no significant effect on quercetin content in *Hypericum attenuatum* Choisy at the early stage, while its effect gradually reached significance later in the treatment period and continued until flowering. Therefore, spraying 2 mg/L naphthalene acetic acid did not have a significant effect on the samples collected at earlier time points but rather had the greatest effect on the samples collected at later time points.

Flavonoids are important secondary metabolites in plants and play important roles in many biological processes of plants as well as in mediating plant responses to biological and abiotic environmental factors. Current studies have found that flavonoids are essentially synthesised in plants through the shikimic acid pathway generating the phenyl propanoids (C6-C3) skeleton and the acetate pathway serving as a building block for polymeric 2- carbon units to synthesize flavonoid [47]. Related studies have found that plant hormones not only regulate plant growth and development, but also participate in the synthesis of flavonoids in plants. Studies have found that gibberellin (GA) can inhibit flavonoids biosynthesis in Arabidopsis through DELLA protein [48].

Additionally, GA treatment also significantly down-regulates the expressions of phenylalanine ammonla-lyase (PAL) and cinnamic acid 4-hydroxylase (C4H) genes, inhibits phenylpropane metabolic pathways, and reduces the levels of key enzymes in the flavonoid synthesis pathways such as chalcone synthase (CHS), flavanone 3-hydroxylase (F3H), and flavonol synchase (FLS) [48, 49]. MYB is an important transcription factor in plants that regulates the biosynthesis of anthocyanins, flavonols, and other flavonoids in plants [50, 51]. Current study found that GA signaling pathway affects the mRNA transcription level of SG7MYB and reduces SG7MYB gene expression [48]. Abscisic acid (ABA), another natural hormone synthesized by plants, plays an important role in promoting abscission, causing stomatal closure and inhibiting growth. Studies have found that ABA can up-regulate the expression of PAL, C4H, CHS, chalcone lsomerase (CHI), and other key enzyme genes in plants, enhance phenylpropane metabolic pathway, and promote the synthesis of flavonoids [49, 52, 53]. In addition, some studies have also found that ABA can promote the expression of MYB-related transcription factors, and activate the binding of MYB to the promoters of downstream key enzyme genes to activate transcription [52, 54–57]. Hamed et al found that when broad bean plants were given cycocel, ABA levels in the plants was increased both before and during flowering [58]. In this study, it was found that certain concentrations of cycocel could increase the contents of rutin and quercetin in the specific organs of *Hypericum attenuatum* Choisy. The reasons for this phenomenon may be that, on the one hand, cycocel, as an antagonist of GA, inhibits the synthesis of gibberellin or improves the level of the negative regulatory factor of gibberellin in plants, which alleviates and eliminates the inhibitory effect of gibberellin on the phenylpropane metabolic pathway, increased the levels of CHS, F3H, FLS and other key enzymes in the pathway of flavonoid synthesis, and activated the expression of related MYB transcription factors, promoted the synthesis and accumulation of related flavonoids such as rutin and quercetin. On the other hand, spraying cycocel on *Hypericum attenuatum* Choisy promoted the accumulation of ABA in the flowering stage. ABA further enhanced the occurrence of phenylpropane metabolic pathway, activated the expression of MYB-related transcription factors, and promoted the synthesis of key enzymes in the downstream CHS, CHI, and other flavonoid synthesis pathways, thus increasing the accumulation of flavonoids. However, the exact reasons remain to be further investigated. In addition, cycocel regulates the balance of source-sink supply, and carbohydrate metabolism [28], which also provides sufficient carbon source substrates for the synthesis of rutin and quercetin in *Hypericum attenuatum* Choisy.

Naphthalene acetic acid, as a synthetic plant growth regulator, has the similar characteristics and efficacy to indoleacetic acid, and is not oxidatively degraded by indoleacetic acid. Besides the basic function of auxin, NAA also has the effects of promoting cell division and growth, enhancing photosynthesis, promoting rooting and accelerating plant growth and development. It is a commonly used plant auxin analogue in production. In sweet cherry, it was found that NAA treatment can increase the content of acetyl-CoA carboxylase complex (ACC) and ABA-glucose ester (ABA-GE), and ACC overexpression can increase the content of malonyl-CoA, providing sufficient substrate for the synthesis of chalcones and flavonoids [59]. While ABA-glucose ester is the main storage form of ABA [59]. The results of Luo et al. showed that auxin treatment could increase the expression of CHS protein and the accumulation of total flavonoids in grape fruits [60]. At the same time, also several studies have shown that the content of indoleacetic acid in a variety of plants was significantly positively correlated with the content of flavonoids in plants [35]. In addition, studies have found that NAA treatment increased the indoleacetic acid content in ginkgo leaves [39]. The results of this experiment showed that 1mg/L and 2mg/L naphthalene acetic acid were excellent in increasing the contents of rutin and quercetin in the flowering stage. This phenomenon may be that NAA treatment increased the ACC, ABA, and CHS protein levels in *Hypericum attenuatum* Choisy. ACC overexpression increased the content of malonyl-CoA. Increased ABA level up-regulates PAL and C4H gene expression, enhances phenylpropane metabolic pathway, increases 4-coumaroyl-CoA levels [61], activated the expression of related MYB transcription factors [52, 57], and enhances the synthesis of flavonoid precursors [62]. All these provided sufficient substrates and enzymes for the synthesis of flavonoids in *Hypericum attenuatum* Choisy, and promoted the synthesis and accumulation of flavonoids by the organism using the substrates.

The main effect of mepiquat chloride in plants is inhibition plant growth by reducing the activity of gibberellin in plants. MCD can inhibit cell elongation, promote chlorophyll synthesis, improve photosynthetic efficiency, and enhance plant stress resistance. Effects are similar to cycocel. The reason for the change in flavonoid content in *Hypericum attenuatum* Choisy may be similar to that of cycocel. In this study, with increasing concentrations of MCD, the contents of rutin, hyperoside and quercetin in the organs of *Hypericum attenuatum* Choisy showed two patterns. One pattern was an initial decrease followed by an increase, and another pattern was a direct increase. There was no clear rule of effect of MCD on the flavonoid content of *Hypericum attenuatum* Choisy, which may be due to differences in the synthesis pathways of the three flavonoids in Hypericum attenuatum Choisy.

Besides being affected by plant hormones, flavonoids also mediate plant responses to environmental factors. It has been found that the synthesis of flavonoids is stimulated by oxidative stress [63–65]. Excessive reactive oxygen species (ROS) are produced in plants during oxidative stress, whereas flavonoids can inhibit the production of ROS and maintain the balance of redox reactions in the body [66]. In this experiment, plants sprayed with naphthalene acetic acid showed different degrees of yellowing, which indicated that spraying with a certain degree of naphthalene acetic acid may destroy the redox balance in *Hypericum attenuatum* Choisy, causing oxidative stress and creating a degree of stress from adversity. Oxidative stress stimulates the synthesis of flavonoids in the body to scavenge damage caused by ROS, and improve the resistance of plants to adversity [67–69]. Wang et al. [70] found that photosynthesis and plant hormones affect flavonoid content. Yao Yuanyuan [71] and Niu [72] et al. found that relatively low temperatures promoted the accumulation of flavonoids in Hypericum perforatum and buckwheat roasted grain tea. In this study, we speculate that CCC, NAA and MCD may reduce the thermosensitivity of *Hypericum attenuatum* Choisy, and that the relatively low temperature and high photosynthesis promoted the synthesis and accumulation of flavonoids. Further research is needed to determine whether CCC, NAA and MCD affect the thermosensitivity of *Hypericum attenuatum* Choisy.

## Conclusion

The contents of rutin, hyperoside, and quercetin in *Hypericum attenuatum* Choisy at the flowering stage were affected by cycocel, mepiquat chloride, and naphthalene acetic acid. A foliar spray of 1 mg/L naphthalene acetic acid at the early growth stage significantly increased the rutin content of *Hypericum attenuatum* Choisy at the flowering stage. A foliar spray of 100 mg/L mepiquat chloride at the early growth stage significantly increased the hyperoside content of *Hypericum attenuatum* Choisy at the flowering stage. A foliar spray of 200 mg/L cycocel at the early growth stage significantly reduced the hyperoside content of *Hypericum attenuatum* Choisy at the flowering stage. A foliar spray of 1 mg/L naphthalene acetic acid at the early growth stage significantly increased the quercetin content at the flowering stage. In addition, the contents of quercetin, rutin, and hypericin in some organs of *Hypericum attenuatum* Choisy were affected by two or more plant growth regulators. It is not clear whether there are synergistic or antagonistic effects among these different growth regulators, and the mechanism of plant action to increase the content of flavonoids in *Hypericum attenuatum* Choisy, which requires further study.

## Supporting information

S1 File This study’s minimal underlying data. (PDF)
