# Supplementary material for: Effects of plant growth regulators on the contents of rutin, hyperoside and quercetin in Hypericum attenuatum Choisy
Source: PLoS One. 2023 May 3;18(5):e0285134. doi: 10.1371/journal.pone.0285134 (PMC10156007; doi:10.1371/journal.pone.0285134)
Supplement: S1 File — (PDF) [file pone.0285134.s001.pdf]

Table 1. Content of rutin in various parts of *Hypericum attenuatum* growth regulator

Treatments	Leaves(mg/g)	Standard error	Stems(mg/g)	Standard
CK	0.078	0.007	0.011	0.001
100C	0.079	0.002	0.023	0.004
200C	0.045	0.001	0.019	0.005
300C	0.067	0.000	0.012	0.001
100S	0.106	0.007	0.017	0.002
200S	0.035	0.007	0.023	0.006
300S	0.116	0.020	0.028	0.002
1NAA	0.126	0.014	0.036	0.001
2NAA	0.050	0.003	0.035	0.006
3NAA	0.091	0.012	0.012	0.001

Table 2. Content of hyperoside in various parts of *Hypericum attenu* plant growth regulator

Treatments	Leaves(mg/g)	Standard error	Stems(mg/g)	Standard
CK	5.167	0.118	2.054	0.019
100C	5.196	0.040	1.994	0.015
200C	4.619	0.198	2.010	0.193
300C	5.120	0.025	1.875	0.011
100S	5.569	0.052	2.311	0.267
200S	5.281	0.019	1.761	0.175
300S	5.474	0.115	2.590	0.064
1NAA	5.203	0.249	2.528	0.023
2NAA	5.071	0.014	1.826	0.037
3NAA	5.227	0.056	1.887	0.028

Table 3. Content of quercetin in various parts of *Hypericum attenuatu* growth regulator

Treatments	Leaves(mg/g)	Standard error	Stems(mg/g)	Standard
CK	0.1187	0.01587	0.0561	0.00575
100C	0.1239	0.02294	0.0249	0.00318
200C	0.1187	0.01345	0.0549	0.00553
300C	0.1558	0.0027	0.0684	0.00121
100S	0.1426	0.00673	0.0647	0.00179
200S	0.0511	0.00405	0.0348	0.0038

300S	0.0748	0.00526	0.0612	0.00111
1NAA	0.1173	0.01822	0.0515	0.00204
2NAA	0.1755	0.01553	0.0405	0.00435
3NAA	0.1067	0.00637	0.0341	0.00164

Table 4. Increased level of ruti

Treatments	Leaves(mg/g)	Standard error	Percentage increase (%)	Stems(mg/g)
100C	0.00060	0.00023	0.77%	0.01230
200C	-0.03390	0.00071	-43.24%	0.00790
300C	-0.01120	0.00032	-14.29%	0.00110
100S	0.02800	0.00695	35.71%	0.00590
200S	-0.04380	0.00711	-55.87%	0.01200
300S	0.03720	0.00510	47.45%	0.01720
1NAA	0.04730	0.00687	60.33%	0.02460
2NAA	-0.02840	0.00344	-36.22%	0.02420
3NAA	0.01260	0.00609	16.07%	0.00070

Table 5. Increased level of hyper

Treatments	Leaves(mg/g)	Standard error	Percentage increase (%)	Stems(mg/g)
100C	0.02840	0.01985	0.55%	-0.05930
200C	-0.54830	0.04948	-10.61%	-0.04360
300C	-0.04750	0.01253	-0.92%	-0.17830
100S	0.40170	0.02620	7.77%	0.25770
200S	0.11380	0.00963	2.20%	-0.29260
300S	0.30670	0.05758	5.94%	0.53640
1NAA	0.03530	0.02493	0.68%	0.47470
2NAA	-0.09680	0.00698	-1.87%	-0.22800
3NAA	0.05940	0.01410	1.15%	-0.16640

Table 6. Increased level of querc

Treatments	Leaves(mg/g)	Standard error	Percentage increase (%)	Stems(mg/g)
100C	0.00520	0.00115	4.38%	-0.03120
200C	0.00000	0.00067	0.00%	-0.00120

300C	0.03710	0.00270	31.26%	0.01230
100S	0.02390	0.00337	20.13%	0.00860
200S	-0.06760	0.00405	-56.95%	-0.02130
300S	-0.04390	0.00526	-36.98%	0.00510
1NAA	-0.00140	0.00091	-1.18%	-0.00460
2NAA	0.05680	0.00777	47.85%	-0.01560
3NAA	-0.01200	0.00319	-10.11%	-0.02200

Choisy treated with plant

Flowers(mg/g)	Standard error
0.040	0.003
0.065	0.008
0.055	0.007
0.039	0.000
0.078	0.011
0.032	0.007
0.071	0.011
0.117	0.020
0.064	0.002
0.064	0.005

***ıatum* Choisy treated with**

Flowers(mg/g)	Standard error
3.848	0.129
4.059	0.118
4.046	0.081
3.807	0.099
4.443	0.177
3.807	0.062
4.157	0.137
4.242	0.089
4.437	0.179
4.318	0.081

***m* Choisy treated with plant**

Flowers(mg/g)	Standard error
0.8695	0.10019
1.1393	0.14257
1.3206	0.03274
0.7066	0.01082
0.5668	0.06258
0.9219	0.10256

1.5699	0.15838
1.6906	0.0441
1.7112	0.08952
1.3676	0.03735

in in *Hypericum attenuatum* Choisy

Standard error	Percentage increase (%)	Flowers(mg/g)	Standard error	Percentage increase (%)
0.00096	112.84%	0.02490	0.00422	62.09%
0.00121	72.48%	0.01460	0.00375	36.41%
0.00109	10.09%	-0.00100	0.00016	-2.49%
0.00182	54.13%	0.03830	0.00553	95.51%
0.00139	110.09%	-0.00850	0.00357	-21.20%
0.00155	157.80%	0.03070	0.00560	76.56%
0.00101	225.69%	0.07700	0.00975	192.02%
0.00142	222.02%	0.02380	0.00238	59.35%
0.00052	6.42%	0.02390	0.00488	59.60%

ɔside in *Hypericum attenuatum* Choisy

Standard error	Percentage increase (%)	Flowers(mg/g)	Standard error	Percentage increase (%)
0.01537	-2.89%	0.21130	0.05892	5.49%
0.01928	-2.12%	0.19870	0.04037	5.16%
0.01132	-8.68%	-0.04080	0.00988	-1.06%
0.05332	12.55%	0.59580	0.05916	15.49%
0.03504	-14.25%	-0.04040	0.01039	-1.05%
0.06415	26.12%	0.30930	0.06860	8.04%
0.02311	23.12%	0.39420	0.04433	10.25%
0.03743	-11.10%	0.58900	0.05960	15.31%
0.02820	-8.10%	0.47020	0.04064	12.22%

:etin in *Hypericum attenuatum* Choisy

Standard error	Percentage increase (%)	Flowers(mg/g)	Standard error	Percentage increase (%)
0.00318	-55.61%	0.26980	0.03564	31.03%
0.00055	-2.14%	0.45110	0.03274	51.88%

0.00121	21.93%	-0.16290	0.01082	-18.73%
0.00179	15.33%	-0.30270	0.06258	-34.81%
0.00380	-37.97%	0.05240	0.01026	6.03%
0.00111	9.09%	0.70040	0.07919	80.55%
0.00204	-8.20%	0.82110	0.04410	94.43%
0.00435	-27.81%	0.84170	0.08952	96.80%
0.00164	-39.22%	0.49810	0.03735	57.29%
